# Construction and validation of a metabolic-associated lncRNA risk index for predicting colorectal cancer prognosis

**DOI:** 10.3389/fonc.2023.1163283

**Published:** 2023-03-31

**Authors:** Quanjun Lin, Zhiqiang Wang, Jue Wang, Ming Xu, Yihang Yuan

**Affiliations:** Department of General Surgery, Tongren Hospital, Shanghai Jiao Tong University School of Medicine, Shanghai, China

**Keywords:** colorectal cancer, metabolism, lncRNA, prognosis, immunity, drug sensitivity

## Abstract

**Background:**

Metabolic reprogramming is one of the most important events in the development of tumors. Similarly, long non-coding RNAs are closely related to the occurrence and development of colorectal cancer (CRC). However, there is still a lack of systematic research on metabolism-related lncRNA in CRC.

**Methods:**

Expression data of metabolism-related genes and lncRNA were obtained from The Cancer Genome Atlas (TCGA). Hub metabolism-related genes (HMRG) were screened out by differential analysis and univariate Cox analysis; a metabolism-related lncRNA risk index (MRLncRI) was constructed by co-expression analysis, univariate Cox regression analysis, LASSO, and multivariate Cox regression analysis. Survival curves were drawn by the Kaplan-Meier method. The ssGSEA method assessed the tumor microenvironment of the sample, and the IPS assessed the patient’s response to immunotherapy. “Oncopredict” assessed patient sensitivity to six common drugs.

**Results:**

MRLncRI has excellent predictive ability for CRC prognosis. Based on this, we also constructed a nomogram that is more suitable for clinical applications. Most immune cells and immune-related terms were higher in the high-risk group. IPS scores were higher in the high-risk group. In addition, the high-risk and low-risk groups were sensitive to different drugs.

**Conclusion:**

MRLncRI can accurately predict the prognosis of CRC patients, is a promising biomarker, and has guiding significance for the clinical treatment of CRC.

## 1 Introduction

Colorectal cancer (CRC) is one of the most common malignant tumors in the world, ranking third in incidence and second in mortality among all malignant tumors (1). Although great efforts have been made to prevent and control the occurrence and development of CRC. However, it is estimated that by 2040, the number of new cases and deaths of colorectal cancer will continue to increase (2). Therefore, there is an urgent need to find new biomarkers as targets for the prevention and treatment of CRC.

It is well known that one of the hallmarks of cancer is metabolic reprogramming (3). Cancer cells adapt various metabolic pathways to meet their own biosynthetic and energy demands (4, 5). The current study shows that two key nutrients, glucose and glutamine, serve to support cancer cell survival and biosynthesis (6). In addition, certain genes or signaling pathways can also drive cancer cells to undergo metabolic reprogramming, resulting in metabolic adaptation (7, 8). For example, p53, a key molecule in cancer, can reprogram glycolysis, lipid metabolism, amino acid metabolism, etc. in cancer cells (9, 10). Mutual crosstalk between HIF1a and c-myc regulates energy metabolism in cancer cells under hypoxic environment, thereby affecting cell proliferation and differentiation (11–13). Given that metabolism plays a crucial role in cancer, it is necessary to systematically explore the key regulators between metabolism and cancer to gain a deeper understanding of the pathogenesis of CRC.

LncRNA is a class of RNA molecules with poor conservation and a length of more than 200 nt. Current research shows that lncRNA is involved in most physiological and pathological processes, including cell proliferation, differentiation, invasion and metastasis, etc. (14). And existing studies have shown that lncRNA is involved in the occurrence and development of most cancers (14–17). More interestingly, lncRNA regulate energy metabolism in cancer (18, 19). At present, there have been many studies involving lncRNA with specific functions to predict the prognosis of CRC. For example, risk models based on lncRNA related to EMT, immunity, and cuproptosis can effectively predict the prognosis of CRC (20–22). However, there is still a lack of effective metabolic-related lncRNA risk models for predicting the prognosis of CRC patients.

In this study, a metabolism-related lncRNA risk model was constructed, which can effectively predict the prognosis of CRC patients. In addition, this risk model can effectively distinguish the tumor microenvironment of patients, which also provides some insights for the selection of immunotherapy and targeted therapy in CRC patients.

## 2 Materials and methods

### 2.1 Data download and processing

The gene expression data, clinical data and prognostic information of CRC patients were obtained from The Cancer Genome Atlas (TCGA, https://portal.gdc.cancer.gov/) database. The TCGA-CRC cohort (including TCGA-COAD and TCGA-READ cohorts) contained 42 normal and 491 cancerous tissues. Samples with missing data were excluded. The genes related to metabolism-related signaling pathways were sorted out from the molecular signature database (MSigDB, https://www.gsea-msigdb.org/), and 948 metabolism-related genes (MRG) were defined. Due to the lack of information on GSTT1 in the expression profile of the TCGA-CRC cohort. Finally, we investigated 947 MRG, which was consistent with previous studies (23). See **Supplementary Table 1** for detailed information.

### 2.2 Metabolism-related lncRNA screening

Firstly, the “Limma” package was used to perform differential analysis on MRG, and the differentially expressed genes affecting the prognosis of CRC were screened out by univariate Cox regression analysis and defined as hub metabolism-related genes (HMRG). The Spearman method was used to obtain HMRG-related lncRNA through co-expression analysis.

### 2.3 Constructing a risk index for metabolic-related lncRNA signatures

Univariate Cox regression analysis was used to screen out lncRNA affecting prognosis, and LASSO regression and multivariate Cox analysis were used to further screen candidate lncRNA to avoid overfitting. The formula for the risk score based on the results of multivariate Cox regression analysis is as follows, MRLncRI = lncRNA_1_ expression * lncRNA_1_ coefficient+ lncRNA_2_ expression * lncRNA_2_ coefficient+…+ lncRNA_n_ expression * lncRNA_n_ coefficient.

The total cohort was randomly divided into a training cohort and a validation cohort at a ratio of 1:1. The Kaplan-Meier (K-M) method was used to draw the survival curves of patients with different risk scores.

### 2.4 Construction of nomogram

A nomogram was drawn using the “regplot” package combined with the metabolism-related lncRNA risk index (MRLncRI) and clinical parameters. ROC curve, c-index and calibration curve are used to evaluate the accuracy and stability of nomogram

### 2.5 Gene set enrichment analysis

GSEA was performed using the “org.Hs.eg.db”, “clusterProfiler” and “enrichplot” packages to explore the enriched signaling pathways in the high-risk and low-risk groups.

### 2.6 Assessment of the tumor microenvironmental landscape

The 29 immune-related marker gene sets were used for single sample Gene Set Enrichment Analysis (ssGSEA) analysis. The GSVA package was used to perform ssGSEA. The 29 immune-related marker gene sets included 13 immune-related terms and 16 immune-related cells.

### 2.7 Immunophenotype score (IPS)

Previous studies have shown that the IPS score can be used to predict response to immunotherapy in cancer patients (24). The IPS scores of CRC patients were downloaded from The Cancer Immunome Atlas database (https://tcia.at/home).

### 2.8 Drug sensitivity analysis

Using the “Oncopredict” package (25) to predict sensitivity to common drugs in CRC patients.

### 2.9 Sample collection, RNA extraction, and RT-PCR

24 pairs CRC samples and matched paracancerous normal tissues were obtained from Tongren Hospital, Shanghai Jiao Tong University School of Medicine. Briefly, total tissue RNA was extracted using RNA-easy isolation reagent (Vazyme, China). RNA was reverse transcribed into stable cDNA using PrimeScript™ RT Master Mix (Takara Bio, Japan), and finally PCR was performed using SYBR-Green qPCR Master Mix (Vazyme, China) and normalized using GAPDH. The primers involved in this study are listed in **Supplementary Table 2**.

### 2.10 Statistical analysis

All analyzes in this study were done on the R language (version 4.1.2). For non-normally distributed continuous variables, the Wilcoxon rank sum test was used for variance analysis. PCR data were analyzed using Student’s t-test. Correlation analysis using the person method. The K-M method was used to draw survival curves. P<0.05 means the difference is statistically significant.

## 3 Results

### 3.1 HMRG screening

**Figure 1:**
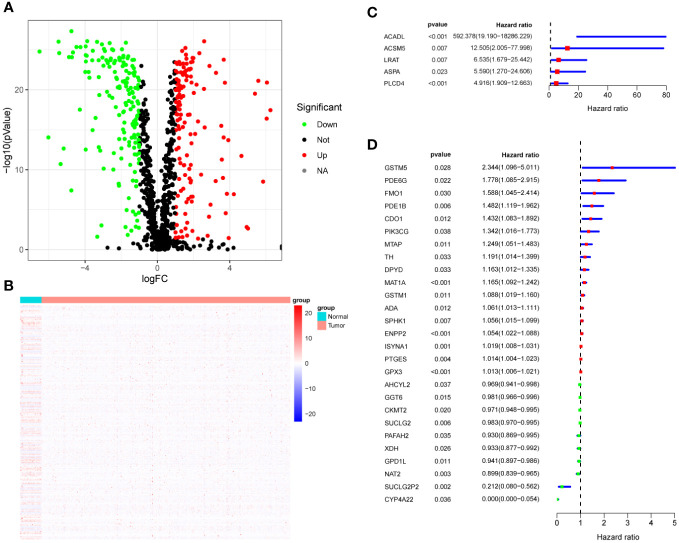
HMRG Screening. **(A)** Volcano plot showing differentially expressed metabolic genes. **(B)** Heatmap showing differentially expressed metabolic genes. **(C, D)** Forest plots showing univariate Cox analysis of DEG survival analysis.

First, we identified 948 MRG and performed differential MRG analysis of in the TCGA-CRC cohort. The results showed that there were 315 differentially expressed genes (DEG), among which 143 MRG were up-regulated and 172 MRG were down-regulated (logFC = 1, p < 0.05). Volcano plots (**Figure 1A**) and heat maps (**Figure 1B**) were used to visualize these results. Then, we performed prognostic analysis on these DEGs by univariate Cox regression analysis, and found that a total of 32 DEGs were prognostic factors in CRC, including 22 risk factors and 10 protective factors (**Figures 1C, D**). The 32 DEGs affecting prognosis were defined as HMRG and further studied.

### 3.2 Construction and validation of MRLncRI

**Figure 2:**
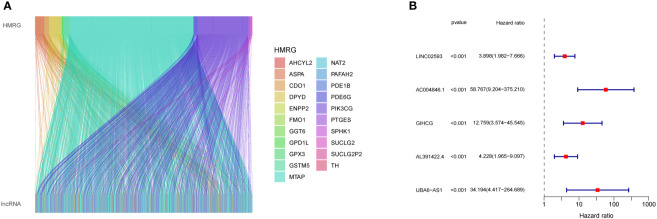
Screening of prognosis-related lncRNA. **(A)** Co-expression network diagram of HMRG and LncRNA. **(B)** Forest plot showing univariate Cox analysis of lncRNA survival analysis.

**Figure 3:**
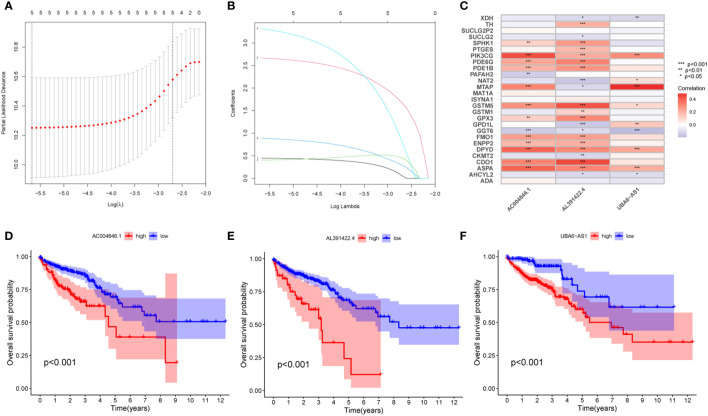
Construction of MRLncRI. **(A, B)** LASSO analysis of prognosis-related lncRNA. **(C)** Correlation heat map of HMRGs and hub lncRNA. **(D–F)** Kaplan-Meier survival curve analysis of hub lncRNA.

First, we performed co-expression analysis of HMRG and lncRNA in the TCGA-CRC cohort. Finally, we obtained 1637 metabolic-related lncRNA (correlation coefficient > 0.4, P < 0.05) (**Figure 2A**). Then, univariate Cox regression analysis obtained 5 lncRNA that were significantly associated with the prognosis of CRC (p < 0.001) (**Figure 2B**). In order to give each patient a scientific quantitative label, we used LASSO regression analysis (**Figures 3A, B**) and multivariate Cox regression analysis (See **Supplementary Table 3** for detailed results) to identify 3 hub lncRNA construction model features, namely AC004846.1, AL391422.4 and UBA6-AS1. Based on the results of multivariate Cox regression analysis, the formula representing MRLncRI was constructed as follows: MRLncRI = AC004846.1 expression * 3.04 + AL391422.4 expression * 1.23+UBA6-AS1 expression * 3.64. We randomly split the TCGA cohort into a training cohort and a validation cohort in a 1:1 ratio. The clinicopathological characteristics of the two groups were first compared and found no statistical difference in the clinicopathological characteristics between the training cohort and the validation cohort (**Table 1**). The correlation of 32 HMRG with 3 hub lncRNA was shown using heatmap (**Figure 3C**).

**Table 1:** Clinicopathological characteristics of the Whole cohort, training cohort, and validation cohort.

Covariates	Type	Whole cohort	Validation cohort	Training cohort	*p* value
Age	<=65	206(43.83%)	100(42.55%)	106(45.11%)	0.6421
	>65	264(56.17%)	135(57.45%)	129(54.89%)	
Gender	FEMALE	219(46.6%)	110(46.81%)	109(46.38%)	1
	MALE	251(53.4%)	125(53.19%)	126(53.62%)	
Stage	Stage I	82(17.45%)	41(17.45%)	41(17.45%)	0.5447
	Stage II	178(37.87%)	84(35.74%)	94(40%)	
	Stage III	125(26.6%)	60(25.53%)	65(27.66%)	
	Stage IV	70(14.89%)	40(17.02%)	30(12.77%)	
	unknow	15(3.19%)	10(4.26%)	5(2.13%)	
T	T1	14(2.98%)	7(2.98%)	7(2.98%)	0.9045
	T2	83(17.66%)	42(17.87%)	41(17.45%)	
	T3	321(68.3%)	160(68.09%)	161(68.51%)	
	T4	51(10.85%)	26(11.06%)	25(10.64%)	
	Tis	1(0.21%)	0(0%)	1(0.43%)	
M	M0	349(74.26%)	176(74.89%)	173(73.62%)	0.1081
	M1	69(14.68%)	40(17.02%)	29(12.34%)	
	MX	45(9.57%)	17(7.23%)	28(11.91%)	
	unknow	7(1.49%)	2(0.85%)	5(2.13%)	
N	N0	276(58.72%)	134(57.02%)	142(60.43%)	0.564
	N1	113(24.04%)	56(23.83%)	57(24.26%)	
	N2	80(17.02%)	44(18.72%)	36(15.32%)	
	NX	1(0.21%)	1(0.43%)	0(0%)	

**Figure 4:**
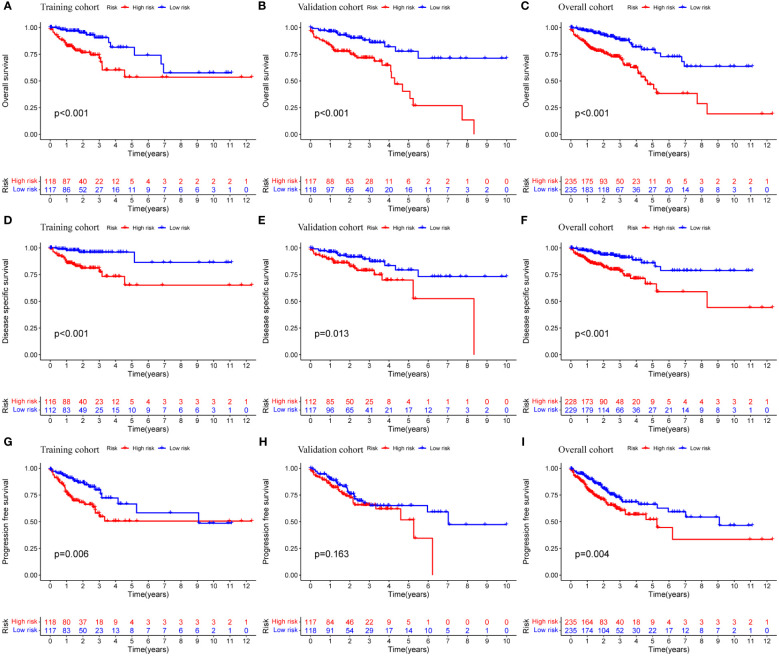
Prognostic analysis of MRLncRI. **(A–C)** Kaplan-Meier survival curve analysis of training cohort, validation cohort and whole cohort OS. **(D–F)** Kaplan-Meier survival curve analysis of training cohort, validation cohort and whole cohort DSS. **(G–I)** Kaplan-Meier survival curve analysis of training cohort, validation cohort and whole cohort PFS.

Prognostic analysis showed that the survival time of patients with low expression of AC004846.1, AL391422.4 and UBA6-AS1 was significantly longer than that of patients with high expression (**Figures 3D–F**). According to the median risk index, the TCGA cohort was divided into high-risk and low-risk groups. Prognostic analysis showed that patients in the high-risk group had significantly lower OS than the high-risk group in the training cohort, validation cohort, and overall cohort (**Figures 4A–C**). In addition, we also evaluated patients’ DSS and DFS, and the results were similar to OS. That is, the DSS and DFS of the high-risk group were significantly shorter than those of the low-risk group in the training cohort and the entire cohort. Although there was no statistical difference in DSS and DFS between the high-risk group and low-risk group in the validation cohort, there was a corresponding trend (**Figures 4D–I**).

**Figure 5:**
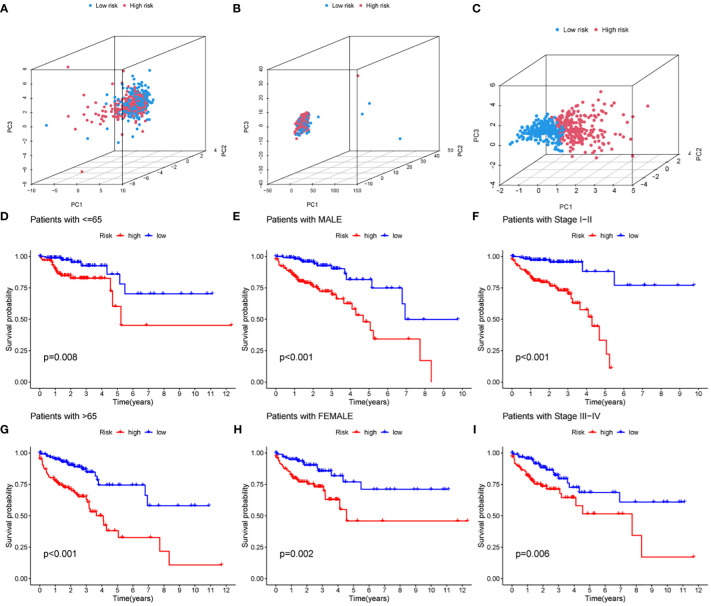
Prognostic analysis of MRLncRI in different pathological features. **(A–C)** PCA analysis of high-risk group and low-risk group based on HMRG, HMRG-related LncRNA and hub LncRNA. **(D, E)** Kaplan-Meier survival curve analysis of high-risk group and low-risk group under different age groups. **(F, G)** Kaplan-Meier survival curve analysis of high-risk group and low-risk group under different gender groups. **(H, I)** Kaplan-Meier survival curve analysis of high-risk group and low-risk group under different tumor stages.

Principal component analysis was performed based on the expression of HMRG, HMRG-associated lncRNA, and hub lncRNA to evaluate the difference between high-risk and low-risk groups. The results showed that the expression of hub lncRNA (**Figure 5C**) but not HMRG and HMRG-related lncRNA (**Figures 5A, B**) could effectively distinguish high-risk and low-risk patients, illustrating the accuracy of the model. In addition, we further discussed the prognostic value of MRLncRI in different clinicopathological feature states. The results showed that the high-risk group had significantly shorter OS than the low-risk group in different ages (**Figures 5D, E**), gender (**Figures 5F, G**) and tumor stage (**Figures 5H, I**).

### 3.3 Nomogram construction based on MRLncRI

**Figure 6:**
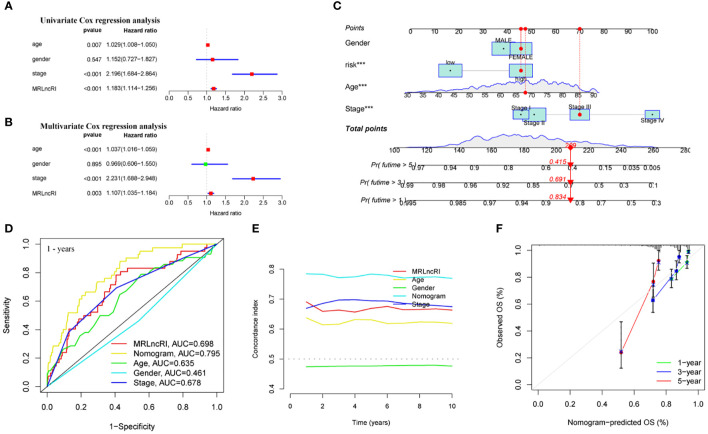
Construction and evaluation of nomogram. **(A)** Univariate Cox regression analysis of MRLncRI and common clinical parameters in CRC. **(B)** Multivariate Cox regression analysis of MRLncRI and common clinical parameters in CRC. **(C)** Nomogram combined with MRLncRI and common clinical parameters. **(D)** ROC of nomogram, MRLncRI and common clinical parameters. **(E)** C-index of nomogram, MRLncRI and common clinical parameters. **(F)** Calibration curves of nomogram predicting 1-year, 3-year and 5-year OS in CRC patients.

We further explored whether MRLncRI is an independent factor affecting the prognosis of CRC patients. We included common clinical parameters (sex, age and stage) for adjustment. Univariate and multivariate Cox regression analysis showed that MRLncRI and tumor stage were independent risk factors affecting the prognosis of CRC patients (**Figures 6A, B**). In addition, in order to highlight the clinical application value of MRLncRI, we constructed a nomogram. The nomogram included MRLncRI as well as three easily accessible clinical parameters (**Figure 6C**). First, we evaluated the sensitivity and specificity of clinical parameters, MRLncRI, and nomogram in predicting patients’ OS using ROC curves. The results showed that the nomogram significantly improved the ability of a single indicator to predict OS (**Figure 6D**). In addition, c-index also shows that nomogram has a strong ability to predict OS (**Figure 6E**). Finally, the calibration curve showed a strong agreement between the predicted and actual values of the nomogram for predicting patient OS (**Figure 6F**). Taken together, these results consistently suggest that the nomogram has excellent potential clinical utility for predicting patient OS.

### 3.4 GSEA

**Figure 7:**
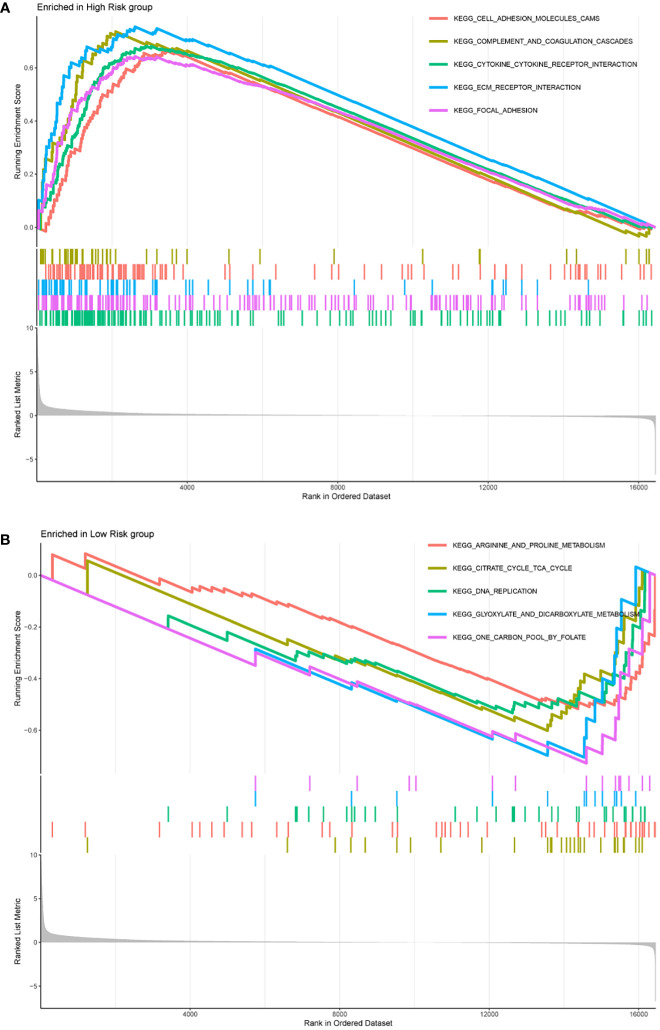
The top five signaling pathways enriched in high-risk group **(A)** and low-risk group **(B)** were shown by GSEA analysis, respectively.

We further used GSEA to explore biological functional differences in different risk groups. The results showed that the high-risk group participated in signaling pathways mainly involving: KEGG CELL ADHESION MOLECULES CAMS, KEGG COMPLEMENT AND COAGULATION CASCADES, KEGG CYTOKINE CYTOKINE RECEPTOR INTERACTION, KEGG ECM RECEPTOR INTERACTION, KEGG FOCAL ADHESION (**Figure 7A**). The signaling pathways involved in the low-risk group mainly involve: KEGG ARGININE AND PROLINE METABOLISM, KEGG CITRATE CYCLE TCA CYCLE, KEGG DNA REPLICATION, KEGG GLYOXYLATE AND DICARBOXYLATE METABOLISM, KEGG ONE CARBON POOL BY FOLATE (**Figure 7B**).

### 3.5 The effect of MRLncRI on the TME and its value in guiding immunotherapy

**Figure 8:**
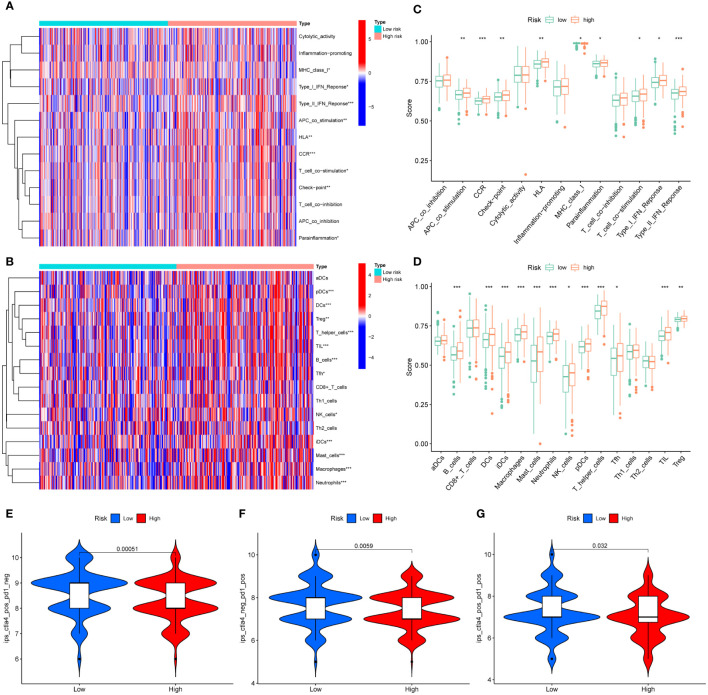
Differences in TME in different risk groups. **(A)** Heatmap of 13 immune-related terms in CRC samples. **(B)** Heat map of 16 types of immune cell infiltration in CRC samples. **(C)** Difference analysis of 13 immune-related terms in high-risk group and low-risk group. **(D)** Difference analysis of 16 kinds of immune cell infiltration in high-risk group and low-risk group. **(E–G)** Difference analysis of IPS scores between high-risk and low-risk groups. * represents p < 0.05, ** represents p < 0.01, and *** represents p < 0.001.

First, we quantitatively scored 13 immune-related terms and 16 immune cell types using ssGSEA and visualized using heatmaps (**Figures 8A, B**). In addition, we conducted a difference analysis, and the results showed that the levels of APC_co_stimulation, CCR, Check−point, HLA, Parainflammation, T_cell_co−stimulation, Type_I_IFN_Reponse, Type_II_IFN_Reponse, B_cells, DCs, iDCs, Macrophages, Mast_cells, Neutrophils, NK_cells, pDCs, T_helper_cells, Tfh, TIL, Treg in the high-risk group were significantly higher than those in the low-risk group (**Figures 8C, D**).

In addition, we further evaluated the guiding value of the MRLncRI for immunotherapy using the IPS. The results showed that the high-risk group had higher IPS scores in the anti-PD1, anti-CTLA4 and combined anti-PD1-CTAL4 treatment groups (**Figures 8E–F**), indicating that patients in the high-risk group may be more suitable for immunotherapy.

### 3.6 Potential drug screening

**Figure 9:**
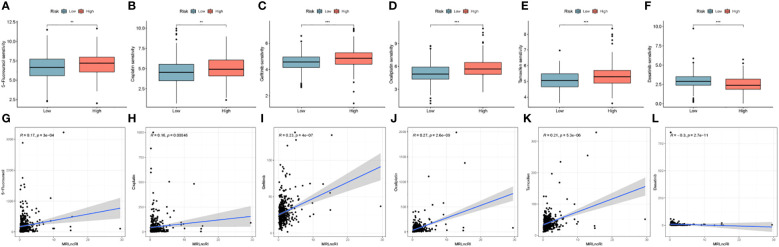
**(A–F)** IC50 difference of 6 common drugs in high-risk group and low-risk group. **(G–L)** Correlation between MRLncRI and IC50 of 6 commonly used drugs. ** represents p < 0.01, and *** represents p < 0.001.

We evaluated the IC50 values of common drugs using the oncopredict package. The results showed that the IC50 values of 5-Fluorouracil, Cisplatin, Gefitinib, Oxaliplatin, and Tamoxifen were lower in the low-risk group, and the IC50 values of Dasatinib in the high-risk group were lower (**Figures 9A–F**). In addition, we also analyzed the correlation between MRLncRI and IC50 using scatterplots (**Figures 9G–L**). The lower the IC50 value, the higher the drug sensitivity and the better the therapeutic effect.

### 3.7 Expression verification of hub lncRNA

This study further explored the expression of 3 hub lncRNA in CRC tissues. The results showed that among the 24 pairs of CRC tissues and adjacent normal tissues, the expressions of AC004846.1, AL391422.4 and UBA6-AS1 in CRC tissues were significantly higher than those in normal tissues (**Supplementary Figures 1A–C**). These results are consistent with those of the bioinformatics analysis.

## 4 Discussion

Nearly a hundred years ago, studies have shown that tumor cells have a significantly higher demand for glucose than normal cells (26), implying metabolic reprogramming of tumor cells. Furthermore, the same phenomenon was found on other tumors as well as metabolites (27, 28). Although the research on metabolism and tumor has been developed for a long time, there is still a lack of biomarkers that can effectively predict the prognosis of CRC. LncRNA participate in various biological processes of CRC, such as growth, metastasis, drug resistance and tumor immune microenvironment (29–31). More importantly, lncRNA can also affect the progression of CRC through metabolic reprogramming (30, 32–34). Therefore, we believe that the systematic analysis of lncRNA related to metabolism is very promising for predicting the prognosis of CRC patients.

In this study, we constructed the MRLncRI by a multidimensional statistical method. Patients were divided into high-risk and low-risk groups according to the median risk index, and the total cohort was randomly divided into a training cohort and a validation cohort. MRLncRI consists of three high-risk lncRNA, AC004846.1, AL391422.4, and UBA6-AS1. Previous studies have shown that UBA6-AS1 can inhibit the proliferation ability of CRC cells *in vitro* and is associated with poorer prognosis (35). This is consistent with the results of this study and also reflects the accuracy of this study. In addition, the prognostic analysis showed that the prognosis of patients in the high-risk group was worse than that in the low-risk group, including OS, DSS, and DFS. In more detail, under different clinical parameters, the prognosis of patients in the high-risk group is worse than that of patients in the low-risk group. More importantly, this study constructed a nomogram that is easier to use clinically. As expected, the ROC, c-index and calibration curves showed that the MRLncRI-based nomogram has high accuracy and robustness in predicting the prognosis of CRC.

Targeting the tumor microenvironment could help improve the effects of immunotherapy, current study suggests (36–38). Tumors with a high degree of immune cell infiltration in the tumor microenvironment and a good response to immunotherapy are called hot tumors, and vice versa are called cold tumors (22). In this study, we estimated the TME of tumor in CRC patients by ssGSEA. The results showed that the level of most immune cells and immune-related terms in the high-risk group was higher than that in the low-risk group. These results mean that patients in the high-risk group tend to have hot tumors and respond better to immunotherapy. Next, we will further explore this view through IPS score. The results showed that the patients in the high-risk group responded better to PD1 and CTLA4-based immunotherapy. In conclusion, the high-risk group is more likely to be classified as hot tumor, and the low-risk group is more likely to be classified as cold tumor.

In addition, drug sensitivity analysis showed that patients in the low-risk group had higher sensitivity to 5-Fluorouracil, Cisplatin, Gefitinib, Oxaliplatin, and Tamoxifen, and patients in the high-risk group had higher sensitivity to Dasatinib, which provided some guidance for the clinical use of CRC patients.

In addition, this study still has some shortcomings. First, no external validation of the prognostic model was performed in this study due to the lack of complete LncRNA expression profiles and prognostic data in public databases. Secondly, in this study, the biological function of the model lincRNA has not been verified by *in vitro* and *in vivo* experiments. In the future, our research will focus more on the exploration of the biological function of the model LncRNA and the external validation of the prognosis model.

## 5 Conclusion

In summary, our study constructed a MRLncRI based on metabolism-related genes and lncRNA. The model can accurately predict the prognosis of CRC patients. In addition, the model can effectively distinguish hot tumors from cold tumors, providing some valuable information for clinical treatment.

## Data availability statement

The original contributions presented in the study are included in the article/**Supplementary Material**. Further inquiries can be directed to the corresponding author.

## Ethics statement

The studies involving human participants were reviewed and approved by The Ethics Committee of Tongren Hospital, Shanghai Jiao Tong University School of Medicine. The patients/participants provided their written informed consent to participate in this study.

## Author contributions

YY were responsible for the design of the study. QL were responsible for data download and analysis and wrote the manuscript. ZW, JW and MX were responsible for picture arrangement and typesetting. All authors contributed to the article and approved the submitted version.

## Supplementary material

Supplementary Figure 1 Expression of hub lncRNA in CRC tissues. **(A–C)** Differential expression analysis of AC004846.1, AL391422.4 and UBA6-AS1 in CRC tissues and adjacent normal tissues.
