# Supplementary figures and images for: Construction and validation of a metabolic-associated lncRNA risk index for predicting colorectal cancer prognosis
Source: Front Oncol. 2023 Mar 31;13:1163283. doi: 10.3389/fonc.2023.1163283 (PMC10102509; doi:10.3389/fonc.2023.1163283)

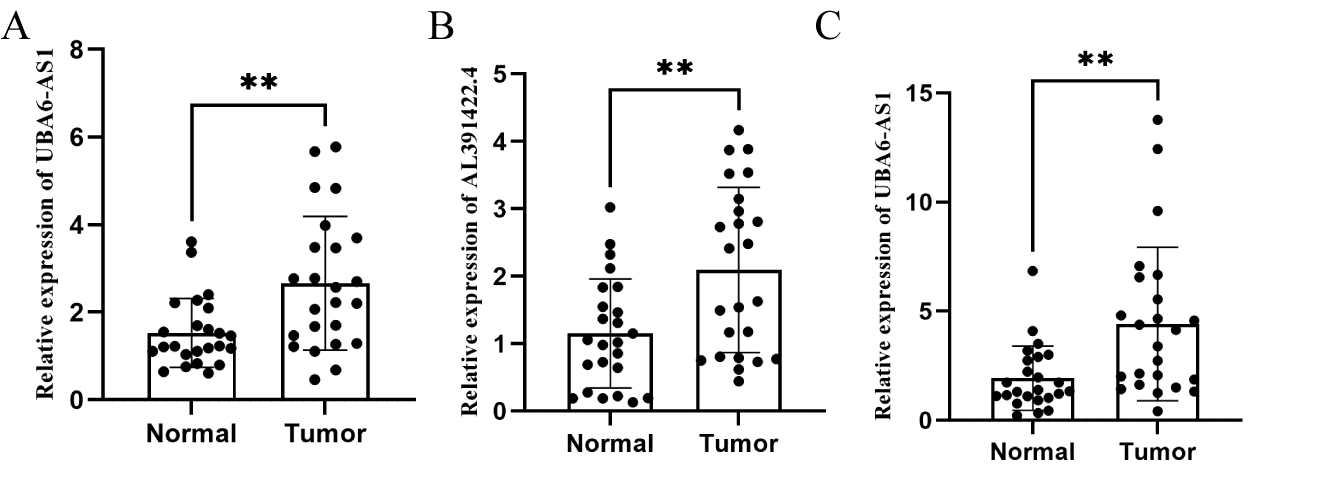

Supplement: Supplementary Figure 1 — Expression of hub lncRNA in CRC tissues. (A–C) Differential expression analysis of AC004846.1, AL391422.4 and UBA6-AS1 in CRC tissues and adjacent normal tissues. [file Image_1.tif]
